# Coalescence of RAGE in Lipid Rafts in Response to Cytolethal Distending Toxin-Induced Inflammation

**DOI:** 10.3389/fimmu.2019.00109

**Published:** 2019-02-26

**Authors:** Hwai-Jeng Lin, Zhi-Pei Jiang, Horng-Ren Lo, Chun-Lung Feng, Chih-Jung Chen, Chia-Yu Yang, Mei-Zi Huang, Hui-Yu Wu, Yu-An Chen, Yu Chen, Cheng-Hsun Chiu, Chih-Ho Lai

**Affiliations:** ^1^Division of Gastroenterology and Hepatology, Department of Internal Medicine, School of Medicine, College of Medicine, Taipei Medical University, Taipei, Taiwan; ^2^Division of Gastroenterology and Hepatology, Department of Internal Medicine, Shuang-Ho Hospital, New Taipei, Taiwan; ^3^Department of Microbiology and Immunology, Graduate Institute of Biomedical Sciences, College of Medicine, Chang Gung University, Taoyuan, Taiwan; ^4^Division of Colon and Rectal Surgery, Department of Laboratory Medicine, Chang Gung Memorial Hospital, Linkou, Taiwan; ^5^Department of Medical Laboratory Science and Biotechnology, Fooyin University, Kaohsiung, Taiwan; ^6^Division of Gastroenterology and Hepatology, Department of Internal Medicine, China Medical University Hsinchu Hospital, Hsinchu, Taiwan; ^7^Department of Microbiology, School of Medicine, China Medical University, Taichung, Taiwan; ^8^Department of Pediatrics, Molecular Infectious Disease Research Center, Chang Gung Memorial Hospital, Linkou, Taiwan; ^9^Department of Nursing, Asia University, Taichung, Taiwan

**Keywords:** RAGE, HMGB1, cytolethal distending toxin, lipid rafts, inflammation

## Abstract

The receptor for advanced glycation end products (RAGE) interacts with various molecules in the cell membrane to induce an inflammatory response. The cytolethal distending toxin (CDT) produced by *Campylobacter jejuni* contains three subunits: CdtA, CdtB, and CdtC. Amongst, CdtA and CdtC interact with membrane lipid rafts, by which CdtB enters the nucleus to induce pathogenesis. In this study, we first explored the relationships between RAGE, lipid rafts, and inflammation in gastrointestinal epithelial cells exposed to CDT. Our results showed that CDT activated the expression of RAGE and high mobility group box 1 (HMGB1), followed by the recruitment of RAGE into lipid rafts. In contrast, RAGE antagonist inhibited CDT-induced inflammation via the RAGE-HMGB1 axis. Disruption of lipid rafts decreased CDT-induced downstream signaling, which in turn attenuated the inflammatory response. Furthermore, *in vivo* studies revealed severe inflammation and upregulation of RAGE and IL-1β in the intestinal tissues of CDT-treated mice. These results demonstrate that mobilization of RAGE to lipid rafts plays a crucial role in CDT-induced inflammation.

## Introduction

*Campylobacter jejuni* is one of the most common causative agents for diarrhea and gastrointestinal diseases in humans (1). CDT produced by *C. jejuni* is composed of three subunits, CdtA, CdtB, and CdtC, which combine to form a holotoxin with cytotoxic activity (2). Among the three toxin components, CdtA and CdtC are pivotal for attachment to the cell membrane, allowing CdtB to enter the cells by endocytosis and to eventually reach the nucleus (3). Nuclear translocation of CdtB, which possesses DNase I activity and induces DNA double-strand breaks (DSB), arrests the cell cycle at the G2/M checkpoint, resulting in cell distention and death (4).

RAGE is a multi-ligand pattern-recognition receptor (PRR), which can interact with advanced glycation end products (AGEs), HMGB1, nucleic acids, and S100 protein family to trigger an inflammatory response (5). Binding of HMGB1 to RAGE activates mitogen-activated protein kinases (MAPKs) and stimulates nuclear factor kappa B (NF-κB), resulting in the release of several proinflammatory cytokines (6, 7). Clinical studies indicated that RAGE plays a crucial role in the development of inflammatory diseases, such as rheumatoid arthritis (8), diabetes mellitus (9), atherosclerosis (10), and inflammatory bowel disease (11). Importantly, RAGE has been implicated in bacterial diseases that contribute to the severity of disease progression (12–14). Although the interaction of HMGB1 and RAGE is correlated with the inflammatory response (15), the mechanism by which CDT regulates RAGE and HMGB1 expression and triggers pro-inflammatory cytokine production to promote inflammation in epithelial cells remains unknown.

The major components of lipid rafts are cholesterol, glycosphingolipids, and phospholipids, which are insoluble in cold 1% Triton X-100. Thus, lipid rafts are referred to as detergent-resistant membranes (DRMs) (16). Numerous pathogens, including bacteria (17–19), viruses (20–22), and protozoan parasites (23) exploit lipid rafts for internalization by cells. Lipid rafts also allow the binding of bacterial toxins to the cytoplasmic membrane and enhance their efficient delivery into cells (24). Our previous studies demonstrated that *C. jejuni* CDT-induced pathogenesis depends on the coalescence of lipid rafts (25, 26). However, whether CDT relies on lipid rafts to induce RAGE expression to facilitate inflammation is unknown.

HMGB1, a nuclear protein, is released from activated immune cells and binds to TLR4 that in turn activates macrophage tumor necrosis factor (TNF) release (27). A recent study demonstrated that HMGB1 binds to LPS to form a complex that efficiently delivers LPS into the cytoplasm through RAGE-dependent endocytosis, which then reaches the endolysosomes (28). Subsequently, HMGB1 permeabilizes the lysosomes in the acidic environment and allows LPS access to the cytosol and caspase-11, which is crucial for pyroptosis. These findings indicate that HMGB1 and RAGE provide a particular transport pathway to the cytosol, and cargo molecules may avoid destruction by the lysosomes when accompanied by HMGB1 (29). Although the mechanisms underlying HMGB1-mediated intracellular LPS delivery have been elucidated, the interactions between extracellular HMGB1 and CDT, which is transported via RAGE to enable CdtB to gain access into the nucleus, are unclear.

CdtB, a part of the holotoxin, is endocytosed and finally reaches the nucleus where it exhibits DNase I activity (4). The close association of CDT with lipid rafts has been found to be crucial for toxin-mediated pathogenesis (25, 26, 30); however, the specific molecules that contribute to this interaction remain unknown. In this study, we investigated the role of RAGE in the CDT-induced inflammatory response in gastrointestinal epithelial cells. We further explored whether lipid rafts are involved in inducing RAGE expression and the subsequent signaling in response to CDT-induced pathogenesis.

## Materials and Methods

### Preparation of Recombinant CDT

Recombinant His-tagged CDT subunits were cloned by following the standard protocols as described previously (25). *E. coli* BL21-DE3 containing *cdtA, cdtB*, or *cdtC* expression plasmids, respectively, were induced by 0.5 mM isopropyl β-D-thiogalactopyranoside (IPTG) at 37°C for 4 h. The expression of His-tagged CdtA, CdtB, and CdtC fusion proteins were purified by metal affinity chromatography (Clontech, Palo-Alto, CA) and characterized by SDS-PAGE and western blot analysis.

### Cell Culture

AGS cells (ATCC CRL 1739) were cultured in F12 medium (Invitrogen), MKN-45 cells (JCRB0254; RIKEN Cell Bank, Japan) and HT29 cells (ATCC HTB-38; human colorectal adenocarcinoma) were cultured in DMEM (Invitrogen), COLO205 cells (CCL-222; human colon adenocarcinoma cells) were cultured in RPMI 1640 medium (Invitrogen). Cell were cultured in medium supplemented with 10% fetal bovine serum (HyClone, Logan, UT) and incubated at 37°C in a humid atmosphere containing 5% CO_2_.

### Cell Cycle Analysis

Each recombinant CDT subunit (100 nM) were added in cell culture medium and incubation at 37°C for 30 min to form a CDT holotoxin (31). After one wash with PBS, AGS cells (1 × 10^6^) were untreated or treated with 100 nM CDT holotoxin for 0, 24, 48, and 72 h. The treated cells were washed and fixed with 70% cold ethanol then incubated at −20°C for 2 h and stained with 20 μg/ml propidium iodine (Sigma-Aldrich, Saint Louis, MO) containing 200 μg/ml RNase A. The stained cells were determined by FACScalibur flow cytometry (Becton-Dickinson, San Jose, CA), and the cell cycle distribution was analyzed by using Cell Quest software WinMDI (Verity Software House, Topsham, ME) as described previously (32).

### SDS-PAGE and Western Blot Analysis

Each recombinant CdtA, CdtB, and CdtC was prepared and subjected to 12% SDS-PAGE, respectively. The gel was stained with Coomassie Brilliant Blue R-250 (Amresco, Solon, OH) for further analysis. AGS cells (5 × 10^5^) were exposed to CDT holotoxin with various concentrations for different time durations. The cell lysates were prepared to resolve by 12% SDS-PAGE and transferred onto polyvinylidene difluoride membranes (Millipore, Billerica, MA). Membranes were probed with primary antibodies: RAGE and HMGB1 (Abcam, Cambridge, UK), and β-actin (Santa Cruz Biotechnology, Santa Cruz, CA) at 4°C overnight. The membranes were then incubated with horseradish peroxidase-conjugated secondary antibody (Millipore, Temecula, CA). The proteins of interests were detected using the ECL Western Blotting Detection Reagent (GE Healthcare, Piscataway, NJ) and visualized by using Azure c400 system and AzureSpot Analysis Software (Azure Biosystems, Dublin, CA).

### Immunofluorescence Staining

AGS cells (2 × 10^5^) were seeded on coverslips and treated or untreated with 100 nM CDT holotoxin for 24 h. Cells were then fixed with 4% paraformaldehyde and probed with the primary antibody against RAGE, followed by incubation with Alexa Fluor 488-conjugate goat anti-rabbit IgG (Jackson ImmunoResearch Laboratories, Inc., Cambridge, UK) and CTX-B Alexa Fluor 555-conjugate (Invitrogen, Carlsbad, CA). Nuclei were counterstained with 4′,6-diamidino-2-phenylindole (DAPI; Sigma-Aldrich, Saint Louis, MO, USA) for 30 min. The stained cells were analyzed using a Zeiss LSM 780 confocal microscope (Carl Zeiss, Oberkochen, Germany) with a 63 × oil immersion objective (numerical aperture of 1.4).

### Reporter Activity Assay

AGS cells were co-transfected with 1 μg *NF-*κ*B* or *IL-8*, and pGL3 luciferase reporters by using jetPEI (Polyplus-transfection, Illkirch, France) according to the manufacturer's instructions. pGL-3 luciferase reporter (Promega, Madison, WI, USA) contains a modified coding region for firefly (*Photinus pyralis*) luciferase that was used to optimize for monitoring transfection efficiency. Reporter lysis buffer (Dual-Luciferase Reporter Assay System; Promega, Madison, WI) was added to each well, and the cells were scraped from the dishes. Equal volumes of luciferase substrate were added to the samples and luminescence was detected using GloMax 20/20 luminometer (Promega), as described previously (33).

### Determination of IL-8 Production

IL-8 production was determined by enzyme-linked immunosorbent assay (ELISA) as described previously (34). Briefly, AGS cells were pretreated with 2 μM RAP (Merck Millipore, Billerica, MA), a RAGE antagonist, for 1 h and exposed to 100 nM CDT holotoxin. After incubation for 24 h, the IL-8 concentration was measured by using a sandwich ELISA kit (Invitrogen, Carlsbad, CA), according to the manufacturer's protocol.

### Co-immunoprecipitation (Co-IP) Assay

The protocol was performed according to the manufacturer's instructions (Immunoprecipitation Kit Dynabeads Protein G, Novex Life Technologies), beginning with the addition of 10 μl of anti-HMGB1 antibody (Abcam, Cambridge, UK) or 10 μl of anti-IgG control (GeneTex, Irvine, CA) to create the co-IP bead-complexes. AGS cells were treated with mock or 100 nM CDT for 24 h at 37°C and cell lysates were prepared. Each sample (50 μg) was added to the anti-HMGB1 antibody or mouse IgG-Dynabeads complexes and incubated for 30 min at 37°C. The bound proteins were eluted and analyzed by western blot assay.

### Animal Study

Male BALB/c mice aged 6-weeks-old were purchased from National Laboratory Animal Center (Taipei, Taiwan). Mice were divided into two groups: PBS treated control (*n* = 3) and 2.5 mg/kg CDT alone (*n* = 3). Each treatment was administered by intragastric gavage once every 2 days for a total of 6 injections. After completing the treatment course, the mice were euthanized and the intestinal tissues were prepared for hematoxylin-eosin (H&E) or immunohistochemistry (IHC) staining. The mice were cared for in accordance with the Laboratory Animal Center of Chang Gung University under a protocol approved by the Institutional Animal Care Use Committee (IACUC Approval No.: CGU16-114).

### Statistical Analysis

Statistics analysis comparisons of more than two groups were evaluated using two-way analysis of variance (ANOVA). The *P*-value for ANOVA had statistically significant difference in those groups, and then used *post hoc* test for ANOVA to analyze the results by Tukey's Honestly Significant Difference Test (Tukey's test). A *P*-value of <0.05 was considered statistically significant. The statistical software was the SPSS program (version 12.0 for windows, SPSS Inc., Chicago, IL).

## Results

### CDT Induces RAGE and HMGB1 Expression

**Figure 1:**
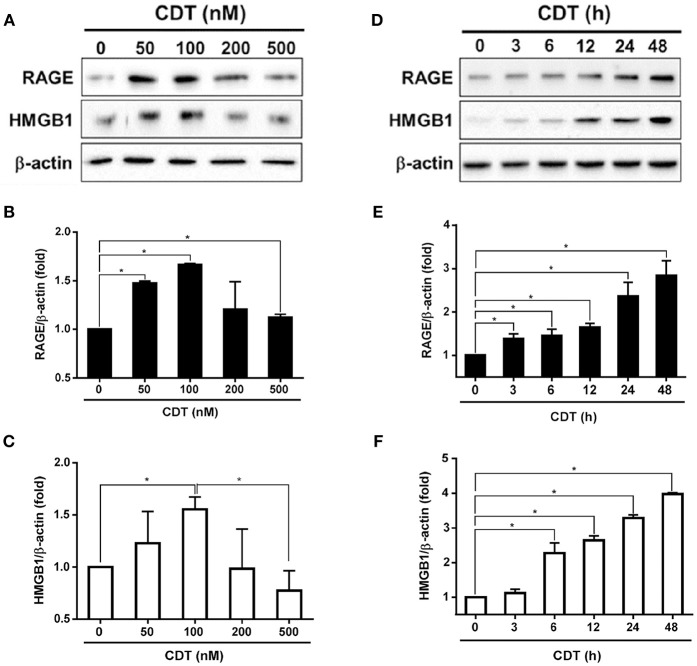
CDT induces RAGE and HMGB1 expression. **(A)** AGS cells were exposed to CDT for 24 h at various concentrations (0–500 nM), and **(B)** treated with 100 nM CDT at different time points (0–48 h). Total cell lysates were prepared to measure the expression of RAGE and HMGB1 by western blotting, and β-actin was used as the protein loading control. Protein expression levels of RAGE and HMGB1 were quantified by densitometric analysis and normalized to β-actin, respectively **(B–F)**. The data are presented as means ± standard deviations for three independent experiments. Statistical analysis was calculated using ANOVA analysis and Tukey's test. ^*^P < 0.05 was considered statistically significant.

Although we previously showed that *C. jejuni* CdtA and CdtC interact with membrane lipid rafts (25), the exact molecules that trigger inflammation are unknown. We therefore established a cell-based assay to determine whether RAGE in lipid rafts contributes to CDT-induced inflammatory signaling. Each His-tagged CDT subunit was purified and validated by SDS-PAGE and western blot analysis (Figure S1). We next examined whether CDT induces cell cycle arrest at G2/M in AGS cells, which is a gastrointestinal-derived cell line. As shown in Figure S2A, treatment of the cells with 50 nM CDT for 24 h caused G2/M arrest in 79% of cells. The percentage of cells arrested at G2/M approached 90% when the concentration of CDT was increased to 100–500 nM. Remarkable cell distention in CDT-treated cells compared to in the CDT-untreated group was observed by light microscopy (Figure S2B). To further examine CDT-induced cell cycle arrest and morphology changes, cells were exposed to CDT holotoxin (100 nM) at 37°C for 0, 24, 48, and 72 h. As shown in Figure S3, the number of cells arrested at G2/M gradually increased and cells became distended upon treating with CDT for 24–48 h. We next investigated whether CDT activated RAGE and HMGB1 expression in the cells. AGS cells were treated with CDT (0–500 nM) for different times, and then RAGE and HMGB1 levels were analyzed by western blotting. As shown in Figures 1A–C, RAGE and HMGB1 expression gradually increased in cells treated with 50–100 nM CDT and slightly decreased upon treatment with 200–500 nM CDT. Additionally, CDT-induced RAGE and HMGB1 expression was markedly increased after incubation with 100 nM CDT for 3–48 h (Figures 1D–F). These results indicate that CDT induced RAGE and HMGB1 expression in dose- and time-dependent manners, and that the optimal conditions were 100 nM CDT and incubation for 24 h. We then investigated whether CDT-induced RAGE and HMGB1 expression in different gastrointestinal-derived cells; four intestinal-derived cell lines (AGS, MKN45, COLO205, and HT29 cells) were employed in this study. Our results showed that the levels of RAGE and HMGB1 were obviously increased in the CDT-treated cells we tested (Figure S4).

### Blockage of RAGE Signaling Decreases CDT-Mediated Inflammatory Response

**Figure 2:**
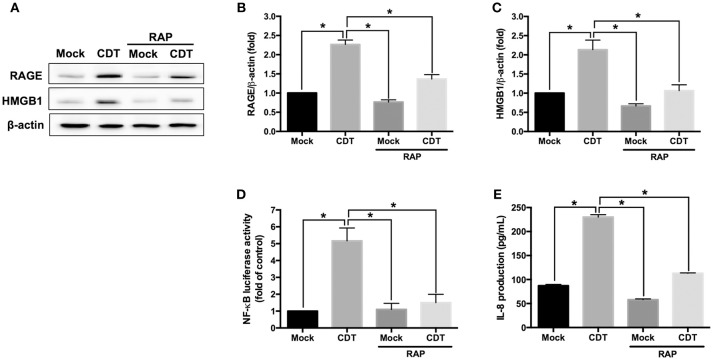
RAGE blockage reduces CDT-mediated inflammatory responses. **(A)** AGS cells were pretreated with RAGE antagonist (2 μM RAP) for 2 h before incubation with 100 nM CDT for 24 h. Cell lysates were analyzed by western blotting with the antibodies against RAGE, HMGB1, and β-actin, respectively. The protein expression of RAGE **(B)** and HMGB1 **(C)** was quantified by densitometric analysis and normalized to β-actin. **(D)** Cells were co-transfected with *NF-*κ*B*- and pGL3-luciferase reporters prior to treatment with the 2 μM RAP followed by exposure to 100 nM CDT for 24 h. pGL3-luciferase reporter was used for monitoring transfection efficiency. *NF-*κ*B* promoter activity was determined and normalized by pGL3 luciferase activity. **(E)** The cell culture supernatant was prepared to evaluate IL-8 production using ELISA. The data are presented as means ± standard deviations for three independent experiments. Statistical analysis was calculated using ANOVA analysis and Tukey's test. ^*^P < 0.05 was considered statistically significant.

The RAGE antagonist RAP, which disrupts the interaction between RAGE and its ligands (35), was employed to investigate whether RAGE is a key factor involved in CDT-mediated inflammation. AGS cells were pretreated with RAP (2 μM) for 2 h prior to treatment with 100 nM CDT, and then the cell lysate was prepared for western blotting. Our results showed that RAP significantly reduced CDT-induced RAGE and HMGB1 expression when compared to CDT treatment alone (Figures 2A–C). We therefore analyzed whether blocking RAGE decreased *NF-*κ*B* promoter activity and IL-8 production in CDT-treated cells. AGS cells were co-transfected with *NF-*κ*B* and pGL-3 luciferase reporters prior to treatment with RAGE antagonist followed by exposure to CDT and were then subjected to luciferase reporter assay. In parallel, culture supernatants were prepared to analyze IL-8 production by ELISA. The results showed that both *NF-*κ*B* promoter activity and IL-8 production were significantly increased in CDT-treated cells, while remarkably decreased in cells pretreated with RAGE antagonist (Figures 2D,E). These results demonstrate that the CDT-induced inflammatory response was mediated through the RAGE signaling pathway.

### CDT Induces the Recruitment of RAGE Into Lipid Rafts

**Figure 3:**
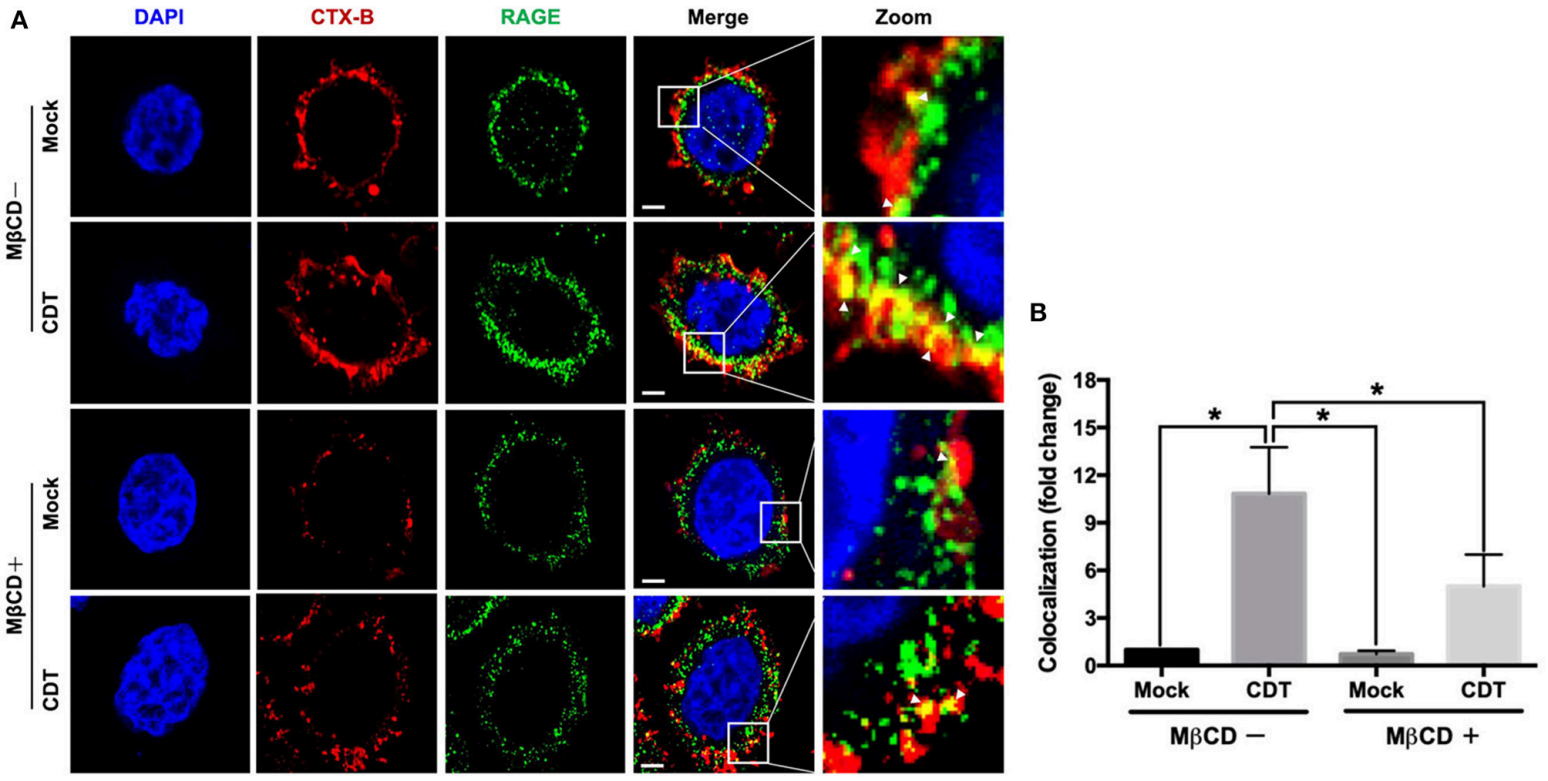
Recruitment of RAGE into lipid rafts by CDT. **(A)** AGS cells were pretreated with or without 5 mM MβCD followed by incubation with 100 nM CDT for 24 h. Cells were fixed and probed with DAPI (blue) to visualize the nucleus, Alexa Fluor 555-conjugated cholera toxin subunit B (CTX-B) to visualize GM1 (red), and an antibody against RAGE (green). Arrows indicated the colocalization (yellow) of CTX-B and RAGE in the overlay. The magnified images were shown in the right panels. Bars, 5 μm. **(B)** The fluorescence intensity of CTX-B and RAGE was analyzed by ZEN software (Carl Zeiss). Colocalized punctate of CTX-B and RAGE were quantified using merged pixels and normalized to those in the mock-control group. Statistical analysis was calculated using ANOVA analysis and Tukey's test. ^*^P < 0.05 was considered statistically significant.

The requirement for lipid rafts to induce RAGE by CDT was evaluated next. As shown in Figure 3A, the colocalization of RAGE with CTX-B (which binds to the ganglioside GM1 in rafts) was clearly localized around the membrane lipid rafts in CDT-treated cells (merged in yellow). However, this colocalization was minimal in CDT mock-treated cells (Figure 3B). We then examined whether the membrane localization of RAGE was dependent on the presence of cholesterol, which is crucial for the composition of lipid rafts. The cells were pretreated with 5 mM methyl-β-cyclodextrin (MβCD, a cholesterol depletion agent) for 1 h and then exposed to CDT holotoxin. As shown in Figure 3, the amount of CDT-induced RAGE that associated CTX-B was visibly reduced upon the cells were pretreated with MβCD. These results suggest that the recruitment of RAGE into membrane rafts occurred in response to CDT treatment.

**Figure 4:**
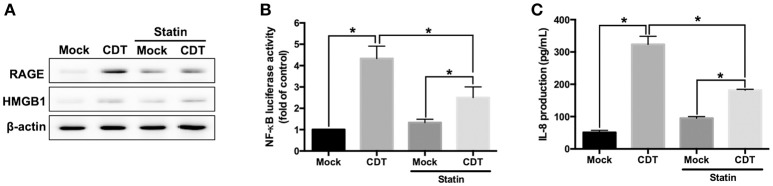
Disruption of lipid rafts decreases CDT-induced inflammatory response. **(A)** AGS cells were pretreated with or without 10 μM lovastatin for 1 h and exposed to 100 nM CDT for 24 h. Cell lysates were analyzed by western blotting with the antibodies against RAGE, HMGB1, and β-actin, respectively. AGS cells were co-transfected with *NF-*κ*B* and pGL3 luciferase reporters in the absence or presence of 10 μM lovastatin before treatment of 100 nM CDT for 24 h. Cell lysates were used to analyze **(B)** *NF-*κ*B* promoter activity and normalized by pGL3 luciferase activity. **(C)** Cell supernatants were subjected to ELISA for the quantification of IL-8 production. The data are presented as means ± standard deviations for three independent experiments. Statistical analysis was calculated using ANOVA analysis and Tukey's test. ^*^P < 0.05 was considered statistically significant.

We next investigated whether CDT-induced RAGE expression and inflammation required membrane raft integrity. AGS cells were pretreated with or without 10 μM lovastatin (an inhibitor of 3-hydroxy-3-methylglutaryl coenzyme A reductase for cellular cholesterol biosynthesis) and then exposed to CDT. As shown in Figure 4A, CDT-induced RAGE and HMGB1 expression were obviously decreased in cells treated with lovastatin. In addition, lovastatin treatment effectively suppressed *NF-*κ*B* promoter activity in CDT-treated cells (Figure 4B). Similarly, CDT-induced IL-8 production was significantly reduced when the membrane cholesterol synthesis was inhibited by lovastatin (Figure 4C). The amount of secreted HMGB1 was then determined by using ELISA. The results showed that both RAP and lovastatin remarkably reduced the secreted HMGB1 in cells treated with CDT (Figure S5). These results demonstrate that depletion of cholesterol inhibited the recruitment of RAGE in lipid rafts and decreased HMGB1 production, which reduced CDT-mediated inflammation.

### CDT Induces Intestinal Inflammation in Mice

**Figure 5:**
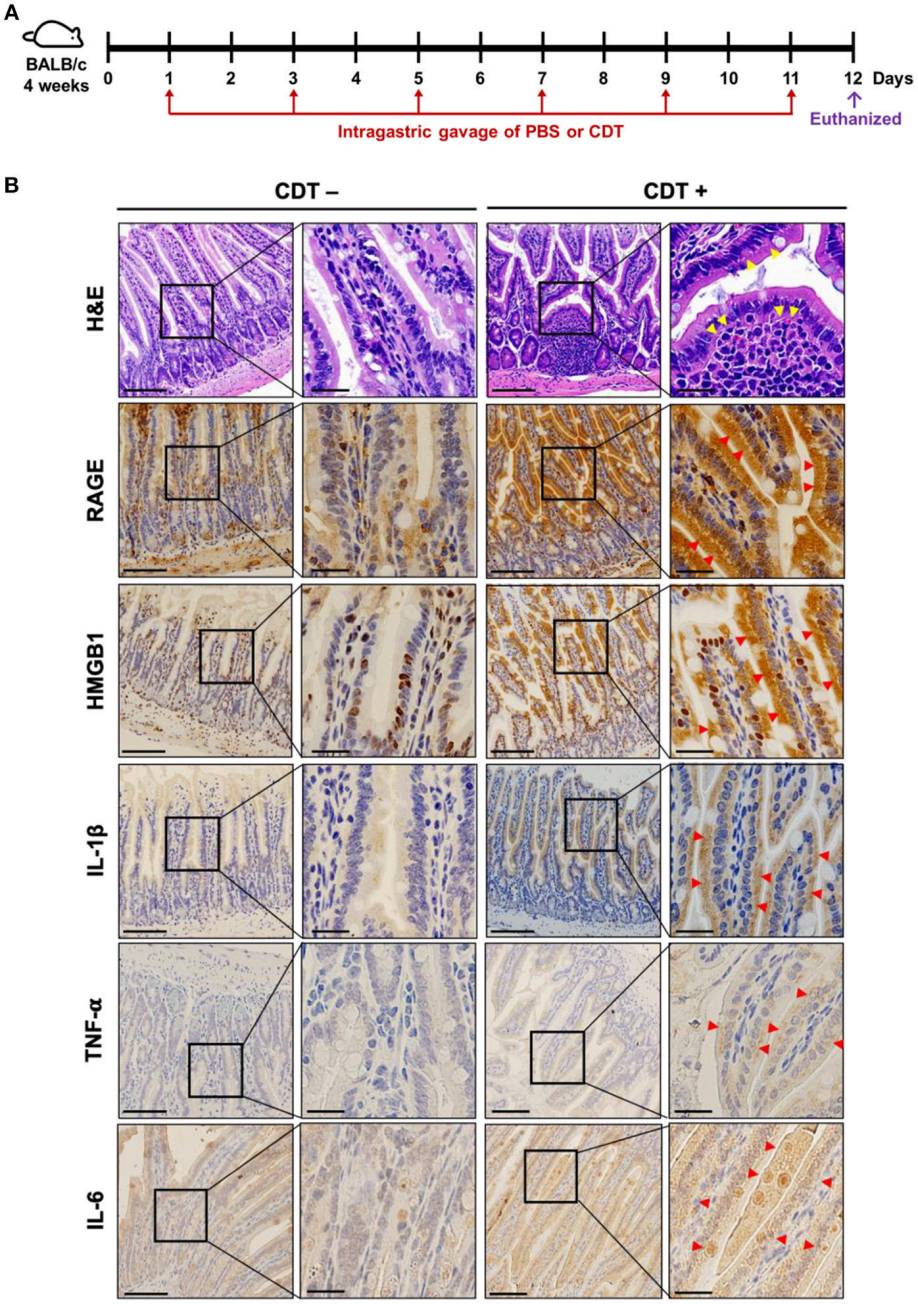
CDT induces RAGE expression and proinflammatory cytokine production in the mouse small intestine. **(A)** Mice were treated with PBS or CDT (2.5 mg/kg) by intragastric gavage once every 2 days for six administrations. Arrows in red indicated the days of CDT administration. **(B)** Tissue sections of the jejunum were prepared and fixed in 4% paraformaldehyde and subjected to hematoxylin-eosin (H&E) or immunohistochemical (IHC) staining with antibodies against RAGE, HMGB1, IL-1β, TNF-α, and IL-6, respectively. The magnified images are shown in the right panel of each cropped area. Arrows in yellow represented severe infiltration of inflammatory cells in the intestinal epithelium with pathological derangement. Pronounced expression of proinflammatory cytokines shown in intestinal tissues were indicated by red arrows. Scale bars in left panels, 20 μm and in magnified right panels, 200 μm.

To further explore the role of RAGE in CDT-mediated inflammation *in vivo*, mice were treated with vehicle-control (PBS) or CDT holotoxin (2.5 mg/kg) through intragastric gavage once every 2 days for a total of six treatments (Figure 5A). After completing the treatment course, the mice were euthanized and tissue sections of the small intestine were prepared for histological analysis. As shown in Figure 5B (H&E staining), the epithelium was clearly defined without inflammation in the intestinal tissues of the vehicle-control. However, pathological examination revealed disruption of the epithelium and severe inflammatory cell infiltration in the intestinal tissues of CDT-treated mice (Figure 5, yellow arrows in the first row). We then examined whether CDT induced the expression of RAGE, HMGB1, IL-1β, TNF-α, and IL-6 in intestinal tissues by IHC. The results revealed stronger expression of RAGE, HMGB1, IL-1β, TNF-α, and IL-6 in the intestinal tissues of CDT-treated mice compared to in the vehicle-control group (Figure 5B). Importantly, HMGB1 was translocated from the nucleus to the cytoplasm upon treatment with CDT. These results, together with those from cell-based and animal studies, demonstrate that RAGE is a crucial factor in CDT-mediated inflammation involving lipid rafts.

## Discussion

RAGE has been reported to participate in several bacterial diseases (14, 34, 36–38). Although RAGE plays a crucial role in inflammation and is required to control bacterial infections, the effect of RAGE on the immune response to CDT has not been investigated. We found that CDT triggers the RAGE-HMGB1-inflammation axis in lipid rafts. Understanding the role of RAGE in CDT-induced pathogenesis is particularly important, as targeting these critical molecules has been proposed for treating bacterial infectious diseases.

The role of RAGE has been investigated by using animal models infected with different bacterial pathogens (14, 36–38), but showed conflicting results. RAGE was found to elevate the burden of *Streptococcus pneumoniae* in the lungs, which exacerbated pneumonia and increased mortality of WT mice compared to RAGE-deficient mice (37). A recent study also reported that RAGE deficiency increased the survival rates of *Acinetobacter baumannii*-infected mice, which was associated with increased levels of circulating IL-10, an anti-inflammatory cytokine (14). In contrast, RAGE deficiency was found to cause organ failure in a mouse model of *E. coli*-induced sepsis, indicating that a RAGE signaling response is involved in its antibacterial activity (36). RAGE contributes to the defense against *Klebsiella pneumoniae* infection by decreasing the bacterial burden and restraining extrapulmonary dissemination, thereby reducing mortality (38). However, the hyperinflammation was occurred in response to gram-negative bacteria by RAGE signaling and that exacerbated the infection in diabetic mice (12).

Consistent with these findings, our study showed that CDT exploited lipid rafts to induce inflammation through the activation of the RAGE-HMGB1-IL-8 axis, indicating that RAGE is a key factor in this process. Notably, the immune defense against pathogen infection is a double-edged sword that either prevents microbial infections or destroys host cells. Therefore, the exact role of RAGE in the beneficial or deteriorated immune defense against CDT-induced pathogenesis requires further investigation.

Danger-associated molecular pattern (DAMP) proteins, such as HMGB1, S100, IL-1α, and IL-33/ST2, are endogenous danger signals (39–41). DAMP signal activation is mediated by several PRRs, including RAGE and Toll-like receptors (TLRs), which are involved in bacteria-induced inflammation (42–44). Several studies have indicated that DAMPs function as alarmins, forming immunostimulatory complexes with chemokines and promoting leukocyte migration and inflammatory responses (15, 45, 46), which are correlated with the severity of bacterial infection (47). RAGE is a ligand for DAMP and is involved in activating NF-κB to stimulate the production of pro-inflammatory cytokines (48). Although we demonstrated that RAGE was mediated during CDT-induced inflammation in the intestine, whether pattern recognition receptors other than RAGE are involved in CDT-induced inflammation is unclear. Identifying mechanisms other than the HMGB1-RAGE interaction is critical for improving the understanding of molecular patterns that occur in response to CDT.

We recently demonstrated that *C. jejuni* CdtA and CdtC interact with membrane-associated lipid rafts, enabling CdtB to cross the cell membrane for transport into the nucleus (25, 26, 30, 49). CdtB possesses DNase I activity, which causes DSBs and leads to cell apoptosis (4). Our current study demonstrate that CDT increased the expression of HMGB1. This can occur at the transcriptional and posttranslational levels, although exactly how the expression of HMGB1 was increased remain unknown. Additionally, it was unclear how CDT influenced HMGB1 to affect the repair of DSB. Despite the availability of genetic information and experimental results, the understanding of CDT-induced pathogenesis at the molecular level warrants further investigations.

HMGB1 is a sticky molecule that binds several proinflammatory molecules including LPS. The HMGB1-LPS complex is endocytosed via RAGE to reach the endolysosomal compartments, then enables LPS to gain access to the cytosol and induce caspase-11 expression, which induces pyroptosis (28). HMGB1 without co-molecules is a strong inducer for cytokines, but it needs TLR4 rather than RAGE for this induction (27, 50). In contrast, HMGB1 with co-molecules can induce cytokines via RAGE. Our study, by using co-immunoprecipitation assay, showed that CDT binds to extracellular HMGB1 that may be important for endocytosis by RAGE (Figure S6A). Although CDT could induce RAGE expression, TLR4 was not involved in this process (Figure S6B). In line with previous studies, our results showed that CdtB and HMGB1 form a complex, which may interact with the cell-surface receptor RAGE. However, whether HMGB1 is essential for translocation of CdtB into the cytosol and finally reaching the nucleus through RAGE-mediated endocytosis require to be investigated.

Although the cell-based assay platform has demonstrated that RAGE plays a crucial role in CDT-induced inflammation, some limitations exist in the current studies, including small number of analyzed mice and did not perform this study in the RAGE or HMGB1-knockout mice. In addition, the direct linkage between RAGE/HMGB1 production and inflammatory response needs to be validated by knockdown or knockout approaches. Further investigations *in vivo* are required to fill in the gap in the translational aspect of the study.

In conclusion, our results demonstrate that RAGE played a crucial role in the CDT-induced inflammatory response. Increased levels of RAGE and HMGB1 were observed in cells treated with CDT. In contrast, RAGE antagonists ameliorated CDT-mediated inflammation by inhibiting the RAGE-HMGB1 axis. Furthermore, disruption of lipid rafts reduced the reporter activities of *NF-*κ*B* and *IL-8* in CDT-treated cells, revealing that CDT-induced inflammation was dependent on lipid rafts. Animal studies further showed that the expression of RAGE and HMGB1, and inflammatory cytokines were increased for the intestinal inflammation in response to CDT. Determining the mechanisms of how CDT triggers inflammation may result in the development of new strategies for controlling bacteria-associated pathogenesis.

## Author Contributions

H-JL, C-HC, and C-HL: conception or design of this work. H-JL, Z-PJ, H-RL, C-LF, C-JC, and C-YY: experimental study. M-ZH, H-YW, Y-AC, and YC: data analysis and interpretation. H-JL, C-HC, and C-HL: writing the manuscript. All authors made final approval.

### Conflict of Interest Statement

The authors declare that the research was conducted in the absence of any commercial or financial relationships that could be construed as a potential conflict of interest.

## Supplementary Material

Figure S1 Characterization of recombinant CDT subunits derived from *C. jejuni*. **(A)** The locations of *cdtA, cdtB*, and *cdtC* on the genome of *C. jejuni* are shown. **(B)** Each CDT subunit (2 μg/mL) was prepared and analyzed using SDS-PAGE. **(C)** Western blot analysis of CDT subunits as detected by antibodies against CdtA, CdtB, or CdtC, respectively. The results represent one of three independent experiments.

Figure S2 CDT induces cell cycle arrest and cell distension. **(A)** AGS Cells were treated with 0–500 nM CDT and incubated at 37°C for 24 h. The cell cycle distribution was analyzed by flow cytometry. **(B)** Cells were exposed to 100 nM CDT for 24 h, and then the cell distention was observed (right panel). Bars, 100 μm. The results represent one of three independent experiments.

Figure S3 CDT arrests cell cycle at G2/M and induces cell distension in a time-dependent manner. AGS Cells were untreated (left panels) or treated (right panels) with 100 nM CDT and incubated at 37°C for 0, 24, 48, and 72 h. The cell cycle distribution was analyzed by flow cytometry and cell morphology was observed by light microscopy. Bars, 100 μm. The results represent one of three independent experiments.

Figure S4 CDT induces RAGE and HMGB1 expression in gastrointestinal-derived cell lines. AGS, MKN-45, COLO205, and HT29 cells were exposed to CDT (100 nM) for 24 h. Total cell lysates were prepared to determine the expression levels of RAGE and HMGB1 by western blotting. β-actin was used as the protein loading control. The results represent one of three independent experiments.

Figure S5 Disruption of lipid rafts and inhibition of RAGE decrease CDT-induced HMGB1 secretion. AGS cells were pretreated with **(A)** RAGE antagonist (2 μM RAP) for 2 h or **(B)** 10 μM lovastatin for 1 h, and then incubated with 100 nM CDT for 24 h. Cell supernatants were subjected to ELISA (G-Biosciences, St. Louis, MO, USA) for the quantification of secreted HMGB1. The data are presented as means ± standard deviations for three independent experiments. Statistical analysis was calculated using ANOVA analysis and Tukey's test. *P* < 0.05 was considered statistically significant.

Figure S6 CdtB binds to extracellular HMGB1 and induces inflammation. **(A)** AGS cells were mock-treated or treated with 100 nM CDT for 24 h and then subjected to co-IP and western blot analysis as described in the Materials and Methods. **(B)** Total cell lysates were prepared to determine the expression levels of TLR4, RAGE, COX-2, and iNOS by western blot assay. β-actin was used as the protein loading control. The results represent one of three independent experiments.
