# Supplementary figures and images for: Coalescence of RAGE in Lipid Rafts in Response to Cytolethal Distending Toxin-Induced Inflammation
Source: Front Immunol. 2019 Feb 26;10:109. doi: 10.3389/fimmu.2019.00109 (PMC6399302; doi:10.3389/fimmu.2019.00109)

## Slide 1
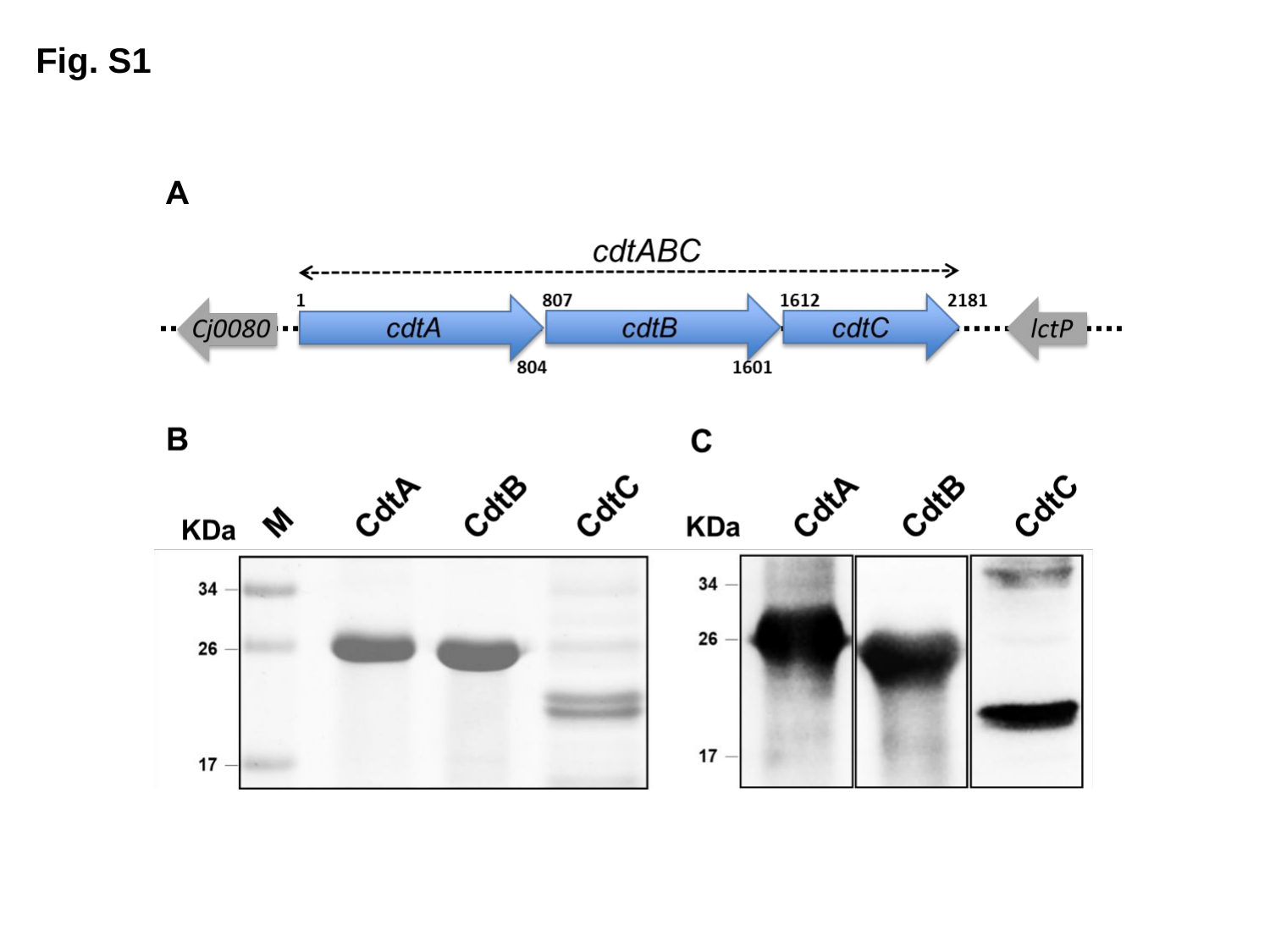

Fig. S1

## Slide 2
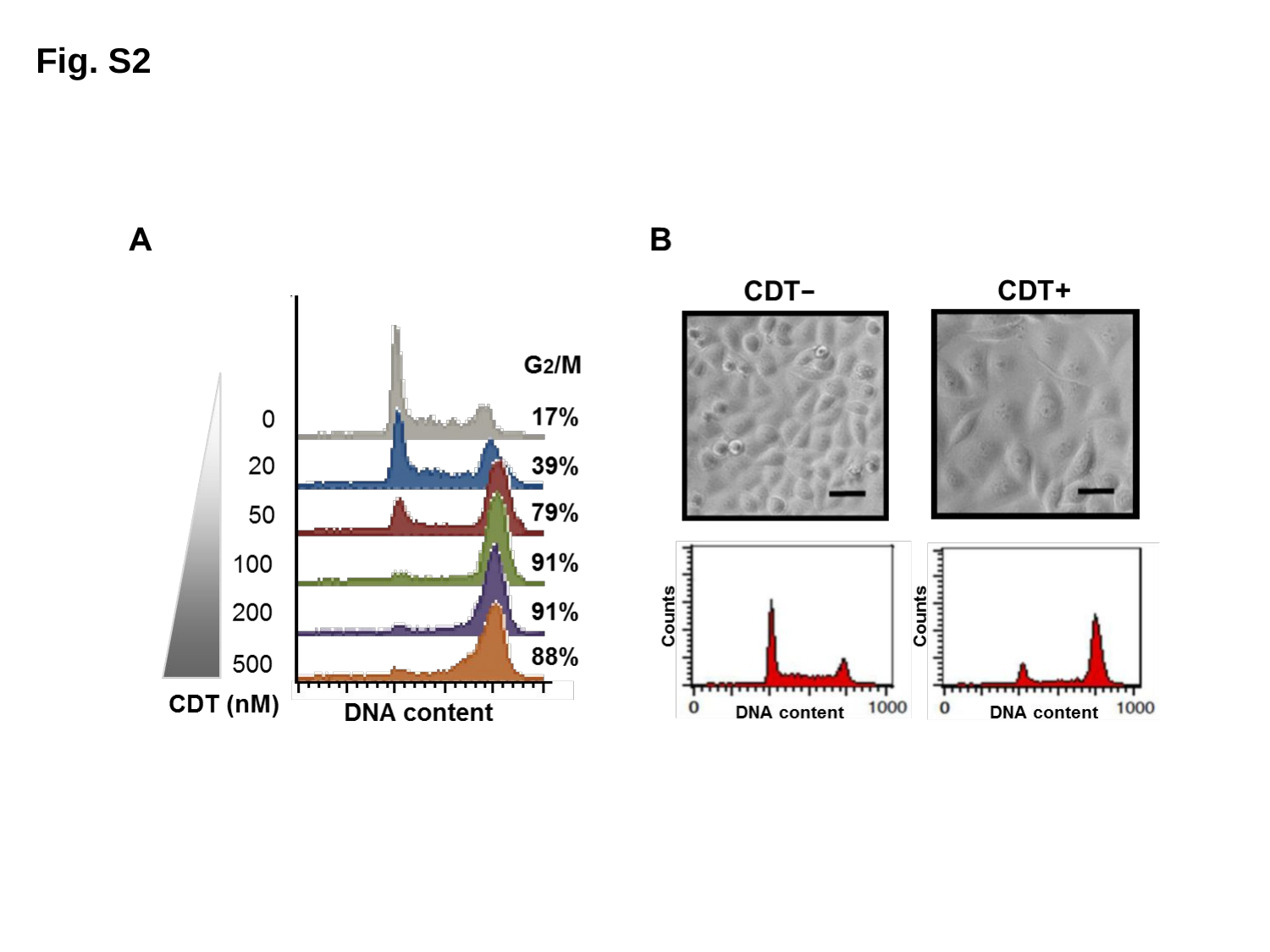

Fig. S2

## Slide 3
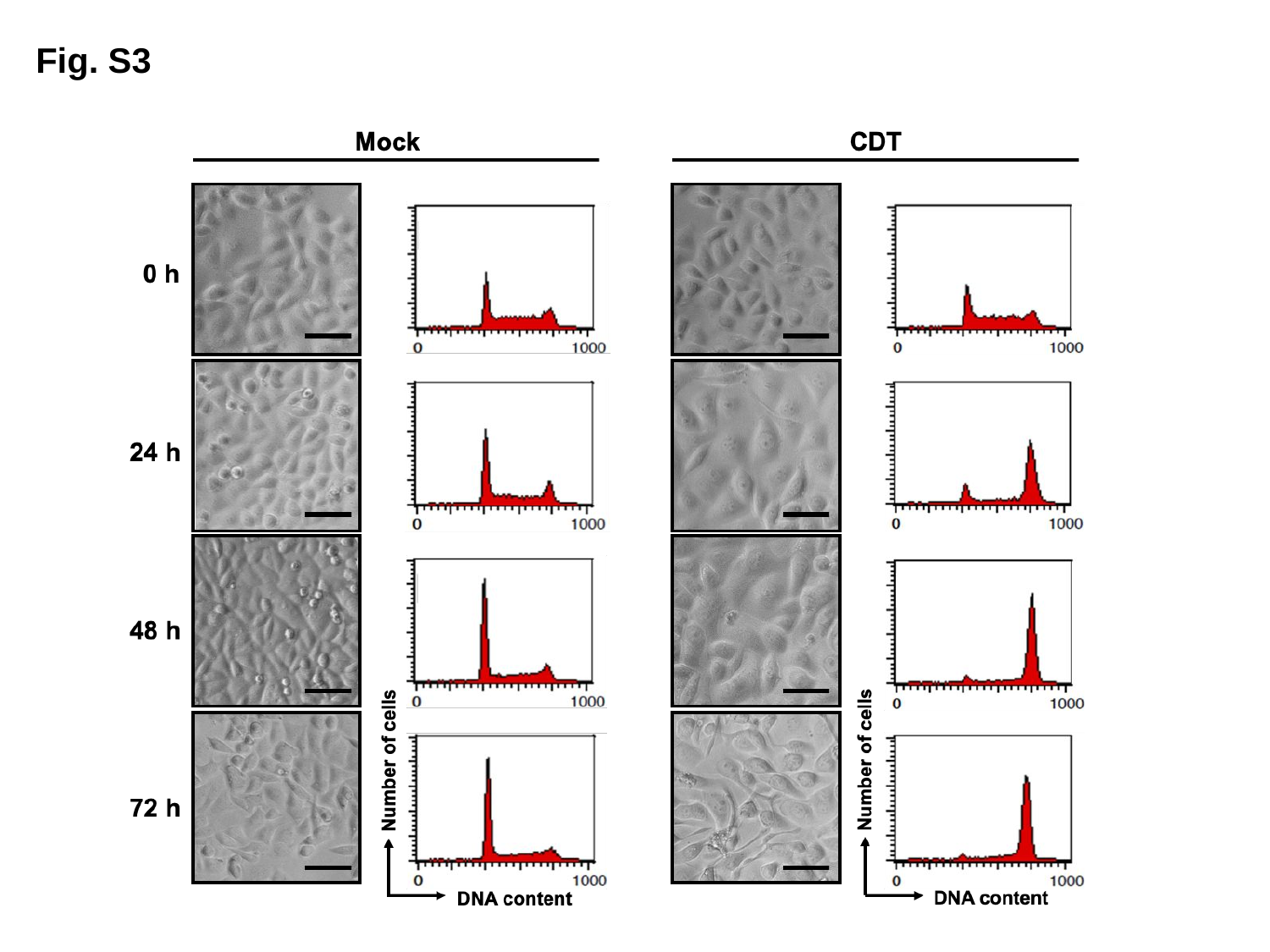

Fig. S3

## Slide 4
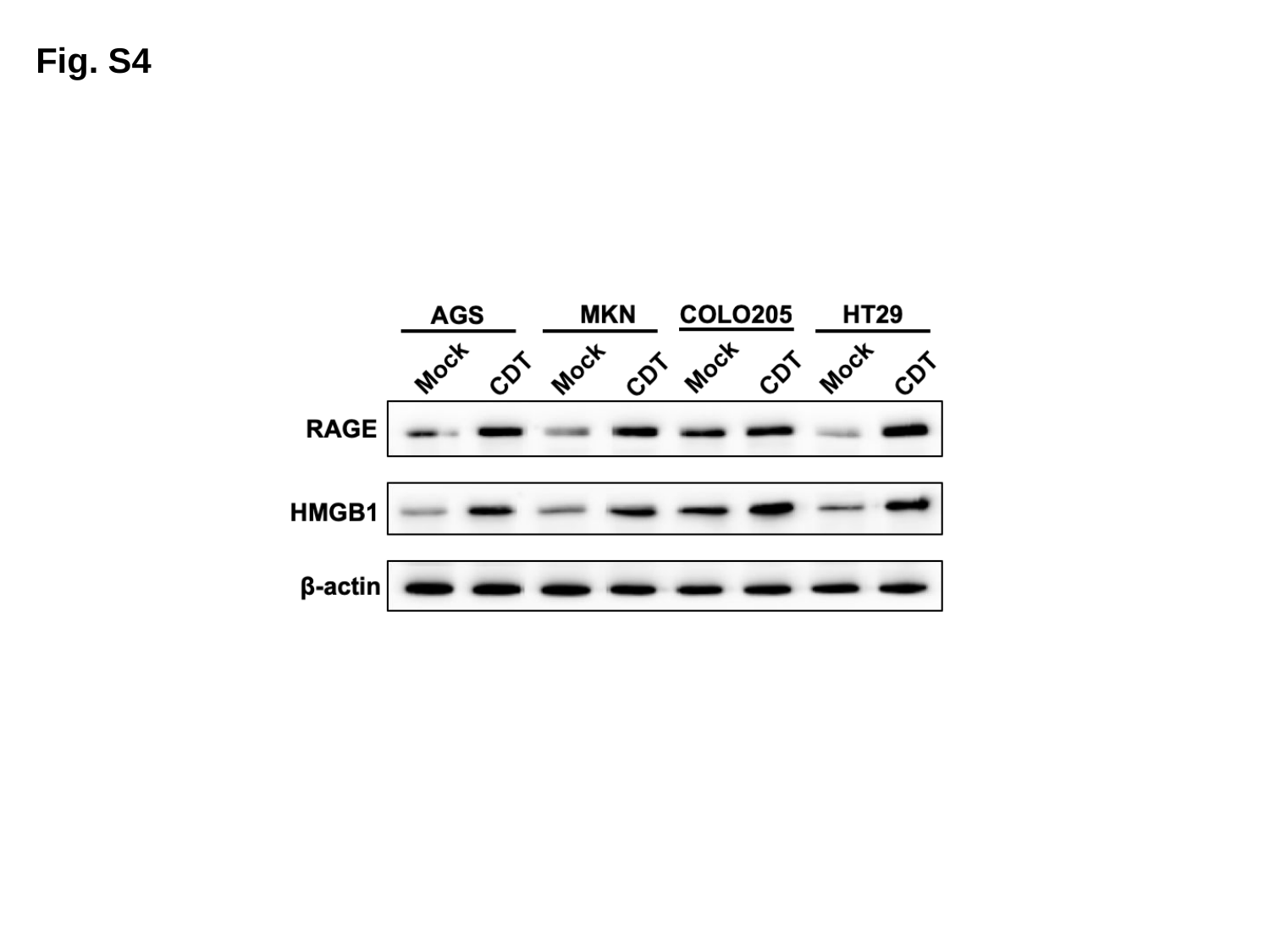

Fig. S4

## Slide 5
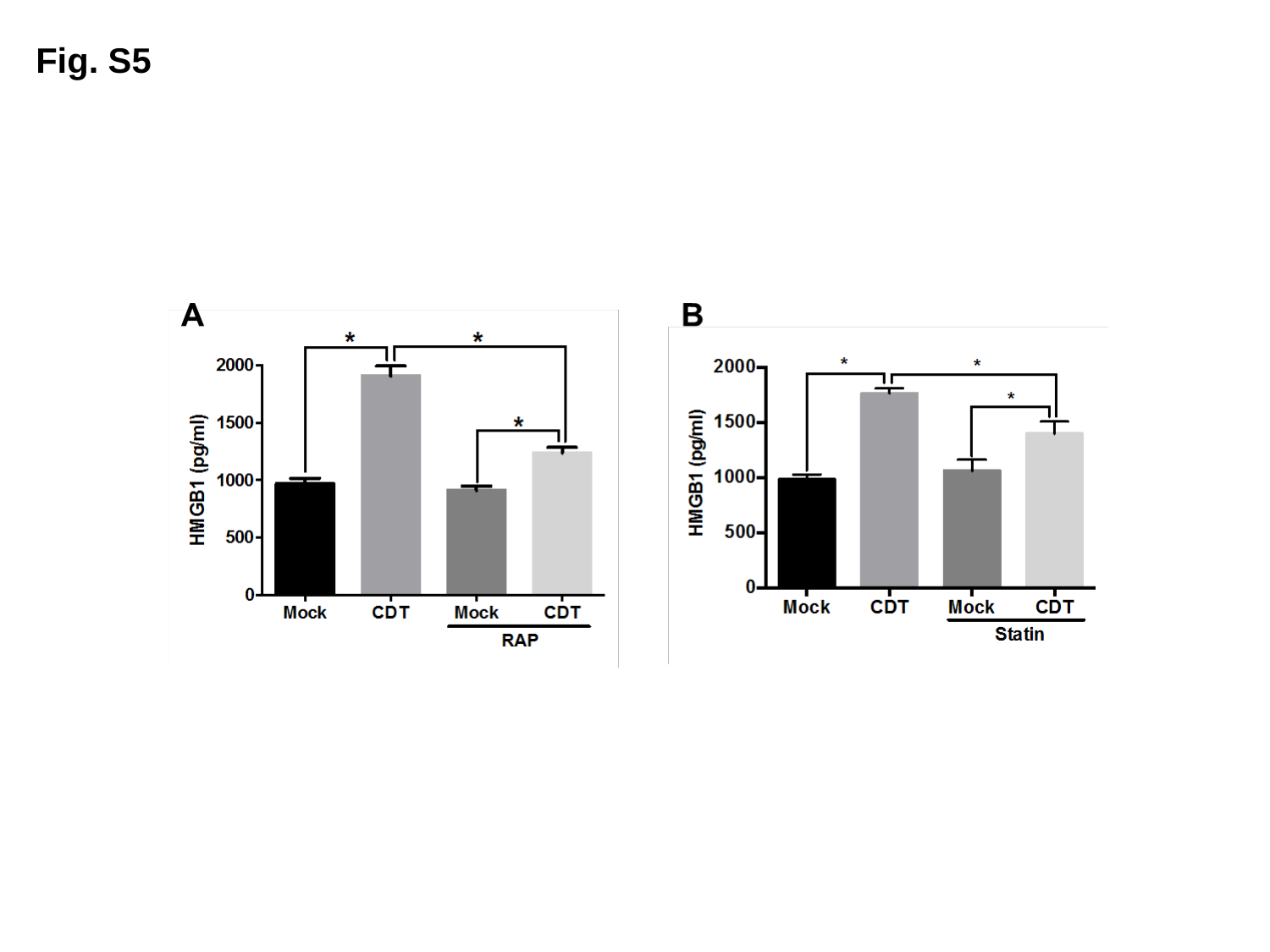

Fig. S5

## Slide 6
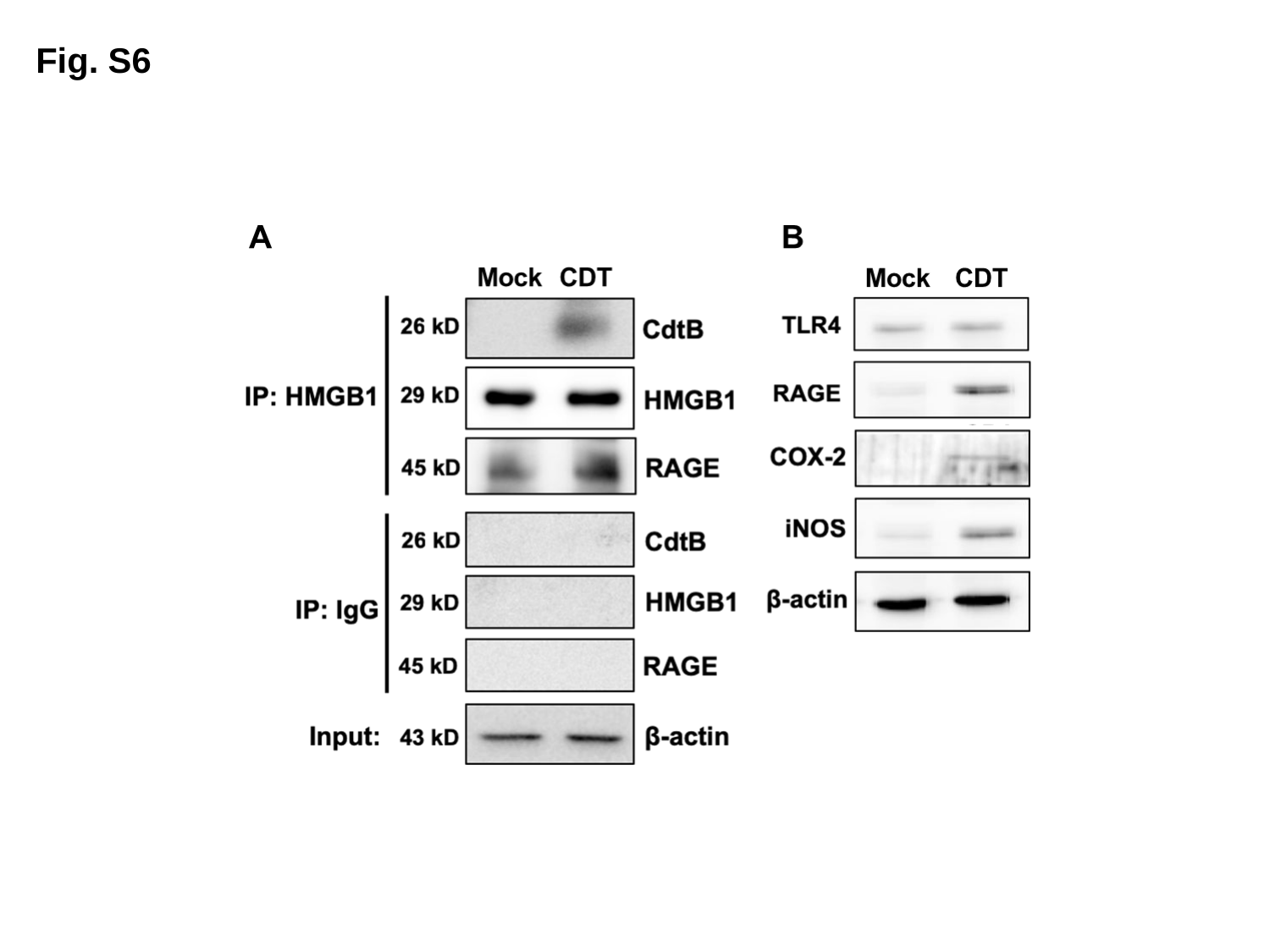

Fig. S6

Supplement: Figure S1 — Characterization of recombinant CDT subunits derived from C. jejuni. (A) The locations of cdtA, cdtB, and cdtC on the genome of C. jejuni are shown. (B) Each CDT subunit (2 μg/mL) was prepared and analyzed using SDS-PAGE. (C) Western blot analysis of CDT subunits as detected by antibodies against CdtA, CdtB, or CdtC, respectively. The results represent one of three independent experiments. [file Presentation_1.PPTX]
